# Editorial: ICT-based training intervention for healthy aging: ITIHA

**DOI:** 10.3389/fphys.2022.1067462

**Published:** 2022-10-28

**Authors:** Achraf Ammar, Khaled Trabelsi, Jordan M. Glenn

**Affiliations:** ^1^ Department of Training and Movement Science, Institute of Sport Science, Johannes Gutenberg-University Mainz, Mainz, Germany; ^2^ Interdisciplinary Laboratory in Neurosciences, Physiology and Psychology: Physical Activity, Health and Learning (LINP2), UFR STAPS (Faculty of Sport Sciences), UPL, Paris Nanterre University, Nanterre, France; ^3^ High Institute of Sport and Physical Education, University of Sfax, Sfax, Tunisia; ^4^ Research Laboratory, Education, Motricity, Sport and Health (EM2S), LR15JS01, High Institute of Sport and Physical Education, University of Sfax, Sfax, Tunisia; ^5^ Department of Health, Exercise Science Research Center Human Performance and Recreation, University of Arkansas, Fayetteville, AR, United States

**Keywords:** physical activity, functional fitness, exercise intervention, active healthy ageing, health information technology (HIT), E-interventions, exergaming, digital healthcare

## Introduction

Currently, physical inactivity is well recognised as one of the most important aging-related risk factors linked to cognitive and motor decline (Norton et al., 2014). Sedentary lifestyle is a primary contributor to the increased rates of overweight and obesity among older adults (Gray et al., 2018), which represent a population at higher risk for motor and cognitive decline due to the adverse effects of oxidative stress, inflammation, vascular, and neuro-muscular function (Keaney et al., 2003; World Health Organization, 2018a). Conversely, physical activity (PA) appears to convey a protective effect against motor and cognitive decline in ageing (George and Reddy, 2019). While the long-term cardiac and cognitive promoting effects of physical exercise are encouraging in healthy older adults (Campbell et al., 2019; Müller et al., 2019), the potential of regular physical training to counteract age-related physical and cognitive declines need to be further confirmed in older adults with existing motor and cognitive decline. Contrarywise, in line with the emerging evidence from the power of digital health solutions to allow easy and accurate characterization and intervention in health and disease (Bhavnani et al., 2016), recent World Health Organisation (WHO) reports indicate that an Active and Healthy Aging (AHA) approach should follow the current revolution in Information and Communication Technology (ICT) (World Health Organization, 2018b). Particularly, WHO highlighted the importance of developing smart solutions/living environments to facilitate the delivery of healthcare services (e.g., physical training program) among healthy and diseased elderly populations. This Research Topic (RT) aims to explore the state of ICT-based physical and/or cognitive training intervention on physical and mental health in healthy and/or diseased elderly and the validation of smart solution/environment to foster AHA. In total, five papers have been published in the present RT “*ICT-based Training Intervention for Healthy Aging: ITIHA*,” which partially cover the current scope of research in this emerging field of research.

## Usability and effectiveness of app-based exercise training in healthy and diseased elderly populations

Unsupervised exercise programs can help overcome transport and high-cost barriers (Schutzer and Graves, 2004) and can be independently integrated into everyday life (Müller et al., 2021); preliminary evidence suggests digitally delivered exercise programs (e- and m-training programs) may provide beneficial impact on physical fitness in the elderly (Jungreitmayr et al., 2021; Netz et al., 2021). In this context, and focusing on healthy retired women, Jungreitmayr et al. examined whether an improvement of physical fitness and associated subdomains (i.e., muscular strength, balance, and flexibility) could be achieved following 14 weeks of an app-based, unsupervised exercise intervention, in which the exercise frequency and duration of sessions were self-selected. Results revealed the adherence (attendance of at least 1.2–2.4 times per week) rate was 71%, which largely exceeded general web-based interventions (average of 50% use at once-weekly). Compared to a control group, the participants who used the app-based physical exercise program had significant beneficial effects on muscle strength and flexibility. While these participants maintained their performance during isometric muscular strength tests and increased their flexibility following the intervention period, the control group decreased in similar parameters. This finding confirms the above-mentioned preliminary evidence and indicates an unsupervised app-based physical exercise program positively influences physical fitness components in women over 60 years of age, notably isometric muscular strength, and flexibility.

Considering the preliminary assumption that PA interventions can alleviate the disease trajectory for individuals with dementia (IWD) (Brasure et al., 2018), information and communication technology (ICT) provides new opportunities to sustainably improve everyday life of IWD in nursing homes. Barisch-Fritz et al. evaluated the usability and effectiveness of ICT-based InCoPE-App in IWD over 65 years old and evaluated the user experience of nursing assistants as well as the trends toward the effectiveness of the digitally delivered intervention on IWD’s physical and cognitive performance. The scores of three questionnaires focusing on different aspects of user experience were higher than reference values (which are available for PSSUQ and ISONORM 9241/110-S), indicating the user experiences of nursing assistants with the InCoPE-App were rated as exceptionally satisfactory. However, the positive user experience of the nursing assistants was not reflected in the intervention’s adherence and training effects. The authors mentioned “the difficult conditions within nursing homes during the COVID-19 lockdowns” as main reason for the high drop-out rate (36.7%), highlighting the need for further research investigating the effectiveness of the InCoPE-App-based exercise intervention, as well as the feasibility and suitability of daily use of the app in a larger sample of IWD.

Diener et al. conducted a systematic review to provide an overview of the effectiveness, acceptability and feasibility of e- and m-Health interventions in terms of promoting PA, preventing falls and positively influencing secondary outcomes, such as physical and cognitive functions, neuropsychiatric symptoms, and psychosocial status in nursing homes (NH). Twenty-eight studies were included in the systematic review involving a total of 1,012 older adults with mean age ranging between 67 and 90 years. Of the 28 studies, 24 contained digital exergaming and four studied e-health intervention, while none incorporated an m-health intervention. The results concluded that E-health/exergaming interventions were feasible and well accepted by healthy NH residents and indicated that the effectiveness of such intervention is influenced by the frequency of weekly training sessions (i.e., ≥ three sessions/week), as opposed to the duration of the intervention. A review of the 21 studies focused on falls and fall risk indicated exergaming mostly led to a significant reduction in the number of falls and fall risk in NH residents, which was not the case for prevention programs delivered *via* videoconferencing. Twenty studies reported secondary outcomes following exergaming, with no available data from the other e-health interventions. Although not consistent across the studies, several significant improvements were also reported on secondary outcomes, but with rare significant differences compared to conventional training. Regarding the impact of exergaming on PA levels of NH residents, no conclusion can be drawn due to the limited number of studies (six studies reported PA-related outcomes with only three of them reporting data on overall PA) and the heterogeneity of the results. Remarkably, most studies excluded individuals with advanced cognitive and physical impairments, and not a single m-health intervention was identified. Therefore, we highlight the urgent need for further research examining the applicability and effectiveness of other digital solutions, besides exergaming, to promote PA in this specific population.

## Feasibility of musical feedback system to enhance PA adherence and mood state in the elderly

Listening to music during exercise has evoked several beneficial effects on physical performance, oxygen consumption, perceived exertion (RPE), and affective valence in different age groups (Terry et al., 2012). In elderly populations, music-based exercise programs improved balance, reaction time and gait, while reducing fall risk (Brown and de Bruin, 2011; Rehfeld et al., 2019). Therefore, developing innovative approaches to combining the benefits of active music-making and physical training, such as the musical feedback systems, has been suggested as a powerful tool to promote active healthy aging. For instance, two systems have been already validated in the adult population, the so-called moBeat to map cyclic movement to music with an ergometer and Jymmin^®^ which synchronize resistance exercise to music. Although the results of the Jymmin^®^ application are promising among young adults in term of force development, oxygen consumption, RPE and mood, this approach has not been tested for older adults. Therefore, the study by Rehfeld et al. evaluated the feasibility of Jymmin^®^ musical feedback system during strength-endurance exercises in 16 healthy older adults (11 females, mean age of 70 years). Compared to conventional workouts, participants exercised significantly longer during Jymmin^®^ music feedback exercise at a constant tempo of 120 beats per minute. However, the RPE and the mental state evaluated by the multidimensional mood state questionnaire did not differ between conventional workout and the Jymmin^®^ conditions. These results suggest Jymmin^®^ can help older adults to exercise longer while expressing similar levels of exhaustions compared to conventional exercise. The authors highlighted the combination of Body Spider and Jymmin^®^ as promising training tools for elderly adults in group settings (in sports clubs, retirement homes, or nursing homes), as well as the need for long-term study in larger populations to confirm their preliminary findings.

## Predicting functional fitness through a multiple linear regression module-based equation

Functional fitness (FF) is defined as an individual’s ability to perform activities of daily living without difficulty, with the main advantage of estimating/tracking the rate of decline in muscle strength, flexibility, agility/dynamic balance, and aerobic endurance with age (Rikli and Jones, 1999). Laboratory methods can accurately measure the FF variables, but are not feasible for testing the entire population due to cost (e.g., sophisticated devices), time, and personal (i.e., qualified technicians) constraints (Kim et al., 2021). Of note is also that older adults with mobility restrictions often have difficulty performing the FF tests. Therefore, the study of Kim et al. aimed to develop a multiple linear regression model for predicting FF variables (e.g., hand grip strength (HGS), lower body strength and flexibility, coordination, agility/dynamic balance, and aerobic endurance) in Korean older adults using easy-to-measure independent variables (e.g., age, sex, body mass index (BMI), and percent body fat). This study analysed data of around 179.000 older adults (>117.000 women), aged above 65 years, acquired from the Republic of Korea’s National Fitness Award (NFA) datasets. In the multiple linear regression model developed, the coefficient of determination of the FF variable was high only in the HGS test regression model [57.708 − (9.602 × sex_male=1; female=2_) + (0.315 × age) − (0.256 × percent body fat) − (0.528 × BMI)] with a mean explanatory power of 77.3% (adjusted *R* ^2^). This HGS mean explanatory power was higher than the ones of previously developed equations in India (52.25%; Mukherjee et al., 2020) and Taiwan [53.3% (Pan et al., 2020)] indicating that the multiple linear regression model formulation by Kim et al. was more accurate and straightforward than the predictive power of previous studies. However, the coefficients of determination in the 30-s chair stand, chair sit-and-reach, figure of 8 walk, timed up-and-go, and 2-min step tests were significantly low to moderate, indicating difficulty in predicting the remaining FF variables (i.e., lower body strength and flexibility, coordination, agility/dynamic balance, and aerobic endurance, respectively) in older adults using easy to measure independent variables. Consequently, the authors encourage future studies to include the physical activity level and nutritional status of older adults when performing similar analyses in order to improve the explanatory power and reach a sufficient level for use in clinical practice and healthcare.

## Concluding remarks

In conclusion, the current published RT studies revealed the usability and applicability of innovative solutions supporting PA interventions such as app-based intervention, exergames, e-intervention, and musical feedback systems. These interventions had significantly higher adherence in older adults compared to conventional interventions. Additionally, most of these studies reported promising findings on the effectiveness of innovative solutions supporting PA intervention in terms of promoting physical fitness and relevant subdomains (i.e., muscular strength, balance, and flexibility), perceived exertion and/or affective valence. Given the limited number of studies on this emerging topic, further large-scale studies in both healthy and diseased older adults are warranted. In future large-scale studies or studies involving older adults with mobility restrictions such as osteoporosis and sarcopenia, the prediction equation of FF, notably the HGS model developed by Kim et al., can be applied for feasibility reasons.
